# An Alternative Model for the Role of RP2 Protein in Flagellum Assembly in the African Trypanosome*

**DOI:** 10.1074/jbc.M113.509521

**Published:** 2013-11-20

**Authors:** Jane Andre, Louise Kerry, Xin Qi, Erica Hawkins, Kristina Drižytė, Michael L. Ginger, Paul G. McKean

**Affiliations:** From the Faculty of Health and Medicine, Biomedical and Life Sciences, Lancaster University, Lancaster LA1 4YQ, United Kingdom

**Keywords:** Centriole, Cilia, Protein Targeting, Trypanosoma Brucei, Tubulin, GTPase-activating Protein, Axoneme

## Abstract

The tubulin cofactor C domain-containing protein *Tb*RP2 is a basal body (centriolar) protein essential for axoneme formation in the flagellate protist *Trypanosoma brucei*, the causal agent of African sleeping sickness. Here, we show how *Tb*RP2 is targeted and tethered at mature basal bodies and provide novel insight into *Tb*RP2 function. Regarding targeting, understanding how several hundred proteins combine to build a microtubule axoneme is a fundamental challenge in eukaryotic cell biology. We show that basal body localization of *Tb*RP2 is mediated by twinned, N-terminal TOF (TON1, OFD1, and FOP) and LisH motifs, motifs that otherwise facilitate localization of only a few conserved proteins at microtubule-organizing centers in animals, plants, and flagellate protists. Regarding *Tb*RP2 function, there is a debate as to whether the flagellar assembly function of specialized, centriolar tubulin cofactor C domain-containing proteins is processing tubulin, the major component of axonemes, or general vesicular trafficking in a flagellum assembly context. Here we report that *Tb*RP2 is required for the recruitment of *T. brucei* orthologs of MKS1 and MKS6, proteins that, in animal cells, are part of a complex that assembles at the base of the flagellum to regulate protein composition and cilium function. We also identify that *Tb*RP2 is detected by YL1/2, an antibody classically used to detect α-tubulin. Together, these data suggest a general processing role for *Tb*RP2 in trypanosome flagellum assembly and challenge the notion that *Tb*RP2 functions solely in assessing tubulin “quality” prior to tubulin incorporation into the elongating axoneme.

## Teaser

**Background:** RP2 function in ciliogenesis is enigmatic.

**Results:** Basal body tethering of *Tb*RP2 depends only on N-terminal TOF-LisH motifs, *Tb*RP2 depletion affects recruitment of transition zone proteins, and *Tb*RP2 encodes the epitope recognized by YL1/2, a monoclonal antibody classically used to detect tyrosinated α-tubulin.

**Conclusion:** The previous model for RP2 function in trypanosomatids is questioned.

**Significance:** We give new insight into the assembly of the ciliary transition zone.

## Introduction

The microtubule axoneme, the defining structure in eukaryotic flagella and cilia, is constructed from several hundred different proteins (1–5). Assembly of this multitude of proteins into a complex three-dimensional architecture is a challenging task, as the flagellum is a spatially distinct compartment in which no protein synthesis occurs. Thus, proteins must be imported into the flagellum compartment from their site of synthesis in the cell body, and, with rare exception (6), flagella grow by elongation at the distal tip of axonemal microtubules. Consequently, tubulin and other axonemal components are transported from the basal body (the canonical microtubule-organizing center that orchestrates flagellum assembly) to the distal tip. This movement is achieved by intraflagellar transport (IFT)^3^ (7), an evolutionary conserved, bidirectional transport mechanism that moves proteins along the axoneme from base to tip (and from tip to base). In a general sense, there is biomedical relevance to understanding flagellum assembly because defects in function are responsible for numerous human genetic syndromes (collectively known as ciliopathies) (8) or can predispose individuals to chronic diseases such as cancer, obesity, and diabetes (9).

Transitional fibers radiating from the basal body demarcate the flagellum as a distinct cellular compartment and define the proximal boundary of the transition zone (TZ), a specialized region at the base of flagella where Y-shaped filamentous connections link axonemal microtubules to the surrounding flagellar membrane (10). In motile flagella, the TZ extends from the transitional fibers to the basal plate, where central pair microtubules are nucleated. Collectively, the transitional fibers and transition zone form the ciliary gate that, in motile and non-motile primary cilia, is proposed to influence flagellar protein content and function (11). The transitional fibers also provide a platform for flagellar protein recruitment, whereupon these proteins are recognized as molecular cargo by the IFT machinery (12).

For the most abundant axonemal protein, tubulin, generation of heterodimeric α/β-tubulin depends upon a canonical folding pathway involving multiple specific tubulin cofactors (TBC). Tubulin cofactor C (TBCC) forms a supercomplex containing α/β-tubulin monomers plus tubulin cofactors D and E and stimulates GTP hydrolysis by β-tubulin, thereby enabling the release of α/β-tubulin heterodimer. It has been proposed that, by ensuring that only the GTPase competent heterodimer is released, cells perform a quality control assessment of heterodimeric tubulin (13–15). In addition to canonical TBCC, most flagellated/ciliated eukaryotes encode another TBCC domain-containing protein, RP2. Biochemical studies on the human RP2 protein, XRP2 (which is mutated in a subset of retinitis pigmentosa patients), suggests that XRP2 and canonical TBCC have a partial functional overlap. XRP2 stimulates GTPase activity of native α/β-tubulin heterodimers but does not generate α/β-tubulin heterodimers from tubulin monomers (16). Restriction of RP2-like proteins to flagellate eukaryotes suggests a specific, and possibly conserved, function for this class of TBCC proteins in flagellum assembly (17). In previous work, we studied RP2 function in the flagellate protist parasite *Trypanosoma brucei*, which is the causal agent of human African sleeping sickness. Although trypanosomes are generally well known for the many unusual aspects of their biology that often impact on virulence (*e.g.* mitochondrial genome organization and RNA editing, antigenic variation, and polycistronic transcription of protein-coding genes (18, 19)), *T. brucei* is an attractive model for studying conserved processes in flagellum assembly because of powerful and tractable reverse genetics. The *T. brucei* RP2 ortholog (*Tb*RP2) is found at the transitional fibers and is required for flagellum assembly (17). On the basis of the range of axonemal defects seen in Tb*RP2* RNAi mutants, the functional overlap between XRP2 and TBCC (16), the colocalization at mature basal bodies of *Tb*RP2::GFP, and the indirect immunofluorescence signal from monoclonal antibody YL1/2 (classically used as a marker for carboxy-tyrosinated α-tubulin), plus loss of YL1/2 labeling following TbRP2 RNAi induction, it was argued that *Tb*RP2 played a critical role in the recruitment/processing of tubulin destined for axonemal incorporation (17).

In contrast, although human XRP2 was initially reported to partially complement a yeast TBCC-deficient mutant (16), it has been suggested more recently to function as a GTPase-activating protein (GAP) for the small ADP ribosylation factor-like GTPase Arl3 and not in a tubulin processing capacity (20–22). XRP2-dependent regulation of Arl3 is proposed to facilitate vesicular trafficking of membrane-associated proteins between the Golgi and the ciliary base, suggesting a more general role in protein processing (20). Thus, notwithstanding very significant differences in the architecture and assembly of the trypanosome flagellum (containing a canonical 9 + 2 microtubule axoneme) *versus* the highly specialized connecting cilium found in retinal cells, there are either critical organismal differences in RP2 function or, at least, debate with regard to the role of RP2 orthologs in flagellum assembly. Moreover, domain architectures of transitional fiber-localized *Tb*RP2, XRP2 (reported to have basal body, flagellar, and Golgi localizations (20, 23–25)), and RP2-like proteins from other organisms (including basal body-localized RPI-2 in the nematode *Caenorhabditis elegans* (26)) differ. For instance, in addition to its TBCC domain, XRP2 contains a degenerate nucleoside diphosphate (NDK) domain at the C terminus and a consensus sequence that specifies for covalent attachment of a myristoyl group to the N terminus, a modification required for targeting XRP2 to the basal body in mammalian cells (23). Both of these features are absent from trypanosome and nematode RP2-like proteins. This not only reinforces the debate regarding RP2 function(s) but also raises the question of how RP2-like proteins are targeted within cells.

Here, we report results from experiments focused on understanding *Tb*RP2 targeting and, in the light of emerging data regarding the composition and role of the ciliary gate, a re-evaluation of *Tb*RP2 function. Our data reveal that basal body targeting and tethering of *Tb*RP2 depends solely upon a combination of N-terminal TOF-LisH motifs, a motif co-option that, for RP2 orthologs, appears to be particular for trypanosomes and their kinetoplastid ancestors, and a motif combination otherwise found in only a few proteins, each of which are found at microtubule organizing centers (MTOCs). Moreover, we also show that recruitment of the ciliary gate components *Tb*MKS1 and *Tb*MKS6 is lost in TbRP2 RNAi mutants and that *Tb*RP2 is itself a target for YL1/2 recognition. With these data, it is likely that the requirement for *Tb*RP2 function in trypanosome flagellum assembly is more complex than the model put forward previously, in which *Tb*RP2 was suggested as a dedicated tubulin folding protein (17).

## EXPERIMENTAL PROCEDURES

### 

#### 

##### Cell Culture and Transfection

Procyclic *T. brucei* (S427 and 927smox (27)) were cultured in SDM-79 medium supplemented with 10% heat-inactivated fetal calf serum and hemin. Logarithmic phase cultures (at densities of ∼5 × 10^6^-10^7^ cells/ml) were stably transformed using standard approaches (28). Selectable markers were used at the following final concentrations: phleomycin, 3 μg/ml (following transfection with p2T7_177_-derived RNAi plasmids); blasticidin, 10 μg/ml (following transfection with pENT6B-derived endogenous tagging plasmids); puromycin, 2 μg/ml (used for routine culture of 927smox); and hygromycin, 50 μg/ml (following transfection with pDEX377-derived expression plasmids). Transgenic cultures were kept free of selectable markers for at least 48 h prior to the start of experiments.

##### DNA Constructs

Fusion proteins were expressed using pEnT or pDEX-based vector systems (29). For expression of GFP::TbRP2 and ^Ty^YFP::TbRP2Δ1–50, DNA sequences corresponding to the partial ORF and 5′ intergenic region were amplified by PCR, and the resultant amplicons were digested with XbaI/XhoI (ORF) or XhoI/BamHI (intergenic region) for ligation into XbaI/BamHI-digested pEnG0 (enhanced GFP) or pEnT6B-Y (YFP). For expression of MKS1::YFP^Ty^ and MKS6::YFP^Ty^, PCR amplicons were digested with SpeI/XhoI (ORF) or XhoI/HindIII (intergenic region) and cloned into SpeI/HindIII-digested pEnT6B-Y. Plasmids were linearized with XhoI prior to transfection. For TbRP2::myc, the DNA sequence corresponding to the *Tb*RP2 open reading frame was synthesized (Eurofins Genetic Services) to encode three tandem-repeated myc epitopes at the C terminus. Codon use was varied to eliminate internal BamHI and HindIII restriction sites from the wild-type Tb*RP2* sequence. The coding sequence of the triple myc tag was codon-optimized for expression in *T. brucei* and separated from the TbRP2 coding sequence by an XhoI site. The start and stop codons of this recombinant gene were flanked by HindIII and BamHI sites, respectively. HindIII-BamHI-digested TbRP2::myc was ligated into pDEX377 that had also been HindIII-BamHI-digested, thereby creating pDEX377_TbRP2::myc_. For expression of TbRP2^Δ418–463^::myc, TbRP2^Δ322–463^::myc, TbRP2^Δ171–463^::myc, TbRP2^Δ134–463^::myc, and XRP2::myc, coding sequences were amplified using a forward primer that contained a 5′ HindIII site and a reverse primer that contained a 5′ XhoI site. PCR amplicons were digested with HindIII and XhoI and ligated into HindIII-XhoI-digested pDEX377_TbRP2::myc_, thereby creating recombinant genes yielding expression of protein with a C-terminal triple-myc epitope tag. For expression of the TbRP2^Δ134–463^::XRP2::myc fusion protein, the 5′ end of the TbRP2 open reading frame (encoding N-terminal amino acids 1–135) was amplified using a forward primer that contained a 5′ HindIII site and a reverse primer that contained a 5′ BamHI site. The human XRP2 open reading frame (minus the ATG start codon) was amplified from a Mammalian Gene Collection RP2 sequence-verified cDNA clone (Thermo Scientific) using a forward primer that contained a 5′ BamHI site and a reverse primer that contained a 5′ XhoI site. PCR amplicons were digested with HindIII-BamHI (TbRP2 N terminus) and BamHI-XhoI (XRP2) and ligated into HindIII/XhoI-digested pDEX377_TbRP2::myc_, creating a recombinant gene that yielded XRP2 protein fused with a TbRP2 N terminus and a C-terminal triple-myc epitope tag. pDEX377-derived plasmids were linearized with NotI prior to transfection. Mutagenesis was performed using the QuikChange site-directed mutagenesis kit according to the instructions of the manufacturer (Agilent Technologies). All plasmids were sequenced using ABI prism sequencing technology (Source Bioscience).

##### Preparation of Recombinant TbRP2 Protein and Anti-TbRP2 Polyclonal Antisera

The *Tb*RP2 open reading frame was amplified by PCR, and the resultant amplicon was digested with NdeI and BglII for ligation into NdeI-BglII-digested pET15b (Novagen), thereby generating a recombinant gene to facilitate expression of *Tb*RP2 protein containing a hexahistidine tag at its N terminus. The resulting plasmid was sequenced using ABI prism sequencing technology (Source Bioscience). The plasmid construct was transformed into *Escherichia coli* BL21 CodonPlus-competent cells (Agilent Technologies), and recombinant protein was induced by addition of 1 mm isopropyl 1-thio-β-d-galactopyranoside. After 3 h of induction at 37 °C, cells were harvested by centrifugation (4500 rpm for 25 min) and lysed by sonication in buffer containing 6 m guanidine, 20 mm Tris-HCl (pH 8), 500 mm NaCl, 0.02% Triton X-100, 20 mm imidazole, and 10% glycerol with 20 mm PMSF. The resulting supernatant was centrifuged at 35000 rpm for 2 h and passed through a 0.45-μm filter. Recombinant His-tagged TbRP2 protein was purified by immobilized metal affinity chromatography and eluted using a linear gradient of 20–500 mm imidazole. Rabbit polyclonal antiserum specific for recombinant *Tb*RP2 was prepared by Eurogentech. For affinity purification, recombinant protein was coupled to CNBr-activated Sepharose (Sigma). Following coupling, the remaining active groups were blocked with 0.2 m glycine (pH 8.1) for 2 h at room temperature, and the coupled affinity resin was then washed with coupling buffer (0.1 m NaHCO_3_ and 0.25 m NaCl (pH8.5)), followed by 0.1 m sodium acetate buffer containing 0.5 m NaCl at pH 4.3. This process was repeated three times before overnight equilibration with PBS (pH 7.7) at 4 °C. Antiserum was diluted at a 1:1 ratio with PBS (pH 7.7) and applied to the resin, and antibodies were eluted under gravity flow with 0.2 m glycine (pH 1.85). The eluate was collected as 1 ml fractions containing 1 m Tris-HCl at pH 8.5 to neutralize the acid. Fractions containing purified antibody were pooled and dialyzed overnight against PBS.

##### Fluorescence Microscopy

Cells were settled onto coverslips and either fixed directly with 3.7% paraformaldehyde or detergent-extracted for 30 s with 1% Nonidet-P40 in 0.1 m PIPES, 2 mm EGTA, 1 mm MgSO_4_, and 0.1 mm EDTA (pH 6.9) prior to paraformaldehyde fixation. Fixed cells were processed further by 10-min incubation in −20 °C methanol followed by rehydration in PBS. Affinity-purified polyclonal antiserum against recombinant *Tb*RP2 was used in indirect immunofluorescence at a 1:50 dilution in PBS-Tween 20 containing 1% BSA. Indirect immunofluorescence with the monoclonal antibodies YL1/2 (30), BBA4 (31), L8C4 (32), and anti-myc was performed as described previously or following the instructions of the supplier (Myc, Abcam). Images were captured using an Applied Precision DeltaVision deconvolution microscope system and processed using SoftWoRx software. Flagella measurements were determined using a Leica DM RXA2 microscope and associated FW4000 software. All images were subsequently processed using Adobe Photoshop.

##### Immunoblotting

Protein samples were separated by SDS-PAGE and immunoblotted onto a Hybond P membrane (Amersham Biosciences) using standard protocols. Membranes were probed with the monoclonal antibodies BB2 (33) to detect MKS1::YFP^Ty^ and MKS6::YFP^Ty^, KMX1 (34) for detection of β-tubulin, and anti-myc (Abcam) for detection of myc-tagged fusion proteins as described previously or according to the instructions of the supplier. Rabbit polyclonal antiserum specific for *Tb*RP2 was used at 1:50 dilution in PBS-Tween 20 containing 5% powdered milk. Detection of HRP-conjugated secondary antibodies was made using Immobilon Western chemiluminescent HRP substrate (Millipore) and a Bio-Rad Chemidoc XRS imaging system and/or Hyperfilm ECL (GE Healthcare).

##### Statistical Analysis

Comparisons of flagellum length between cell populations were statistically evaluated using Minitab 16 software and Mann-Whitney *U* test. Values obtained from this non-parametric procedure were regarded as significant at *p* < 0.005.

## RESULTS

### 

#### 

##### Basal Body Localization of Native and Tagged TbRP2 Variants

**FIGURE 1.**
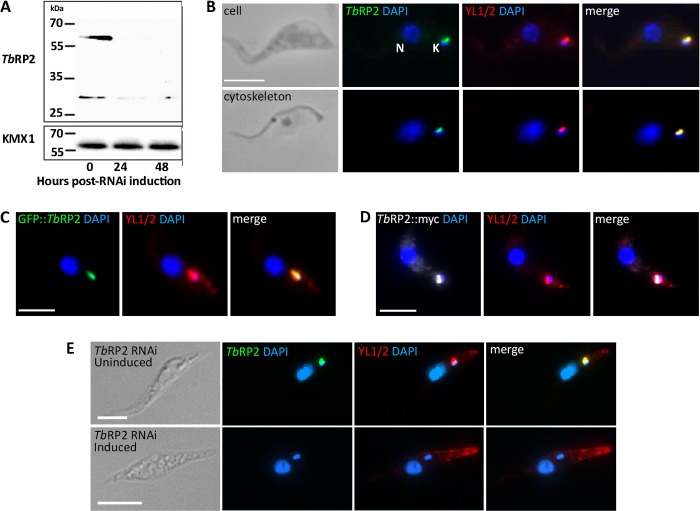
**Localization of native and tagged *Tb*RP2 at mature basal bodies in procyclic *T. brucei*.** *A*, immunoblot analysis reveals that antibodies affinity-purified against recombinant *Tb*RP2 detect protein migrating with an apparent molecular mass slightly higher than the predicted mass of *Tb*RP2 (49.8 kDa). The signal is lost following TbRP2 RNAi induction. A protein of lower molecular mass (∼30 kDa) was also consistently seen in immunoblot analysis. This signal was also lost following Tb*RP2* RNAi induction, suggesting a *Tb*RP2-derived proteolytic cleavage product. The anti β-tubulin monoclonal antibody KMX1 provided the loading control. *B*, indirect immunofluorescence using the monoclonal antibody YL1/2 (classically used to detect tyrosinated α-tubulin) and affinity-purified anti-*Tb*RP2 antibodies in paraformaldehyde-fixed cells and detergent-extracted cytoskeletons. The nucleus (*N*) and mitochondrial genome (kinetoplast, *k*, which is physically attached to the flagellar basal bodies) are indicated in the intact cell panel. *C* and *D*, N- and C-terminally tagged *Tb*RP2 also localize to the mature basal body. *E*, indirect immunofluorescence analysis of Tb*RP2* RNAi cells grown in the absence (*top panel*) or presence (*bottom panel*) of 1 μg/ml doxycycline for 48 h reveals loss of anti-*Tb*RP2 and YL1/2 staining from the mature basal body. YL1/2 detection of tyrosinated α-tubulin within new microtubules at the posterior pole of the cell is retained. *Scale bars* = 5 μm.

To determine how *Tb*RP2 is targeted to and retained on the transitional fibers radiating from mature flagellar basal bodies, we first confirmed that addition of either the C- or N-terminal tags did not affect protein localization. Thus, we raised polyclonal antisera recognizing full-length recombinant *Tb*RP2 and prepared affinity-purified antibodies recognizing the target antigen. Immunoblots of *T. brucei* cell lysates, prepared at various time points after induction of TbRP2 RNAi, showed that affinity-purified antisera recognized full-length TbRP2 (∼50 kDa) and a lower molecular mass band that resolves at ∼30 kDa. The derived fragment most likely represents a proteolytic cleavage product, as it is also depleted following TbRP2 RNAi induction (Fig. 1*A*). Following detergent extraction of intact cells, this smaller peptide partitions into the soluble fraction, whereas full-length *Tb*RP2 partitions into the insoluble cytoskeletal fraction (data not shown). In agreement with previous observations of *Tb*RP2 fused at the C terminus to GFP (17), we showed, by epifluorescence microscopy, mature basal body localizations for native *Tb*RP2 in whole cells and detergent-extracted cytoskeletons (Fig. 1*B*), *Tb*RP2 fused at the N terminus to GFP (GFP::*Tb*RP2) (*C*), and *Tb*RP2 fused at the C terminus to three tandem-repeated myc-epitopes (*Tb*RP2::myc) (*D*). Specificity of the anti-*Tb*RP2 antiserum was also confirmed by loss of the *Tb*RP2 basal body signal in TbRP2 RNAi-induced cells (Fig. 1*E*). The marker for mature basal bodies in these experiments was the monoclonal antibody YL1/2, which is classically used as a marker for carboxyl-tyrosinated α-tubulin in trypanosomes and a wide variety of other eukaryotes (30, 35). In *T. brucei*, in addition to labeling new microtubules forming the subpellicular corset of the cell body (35), YL1/2 has been shown by immunogold EM to label transitional fibers at the distal end of the mature basal body (17). As reported previously (17), induction of TbRP2 RNAi led to the loss of both *Tb*RP2 and YL1/2 labeling at mature basal bodies but had no effect on YL1/2 labeling of subpellicular microtubules (Fig. 1*E*).

##### Targeting and Tethering of TbRP2 at Mature Basal Bodies

**FIGURE 2.**
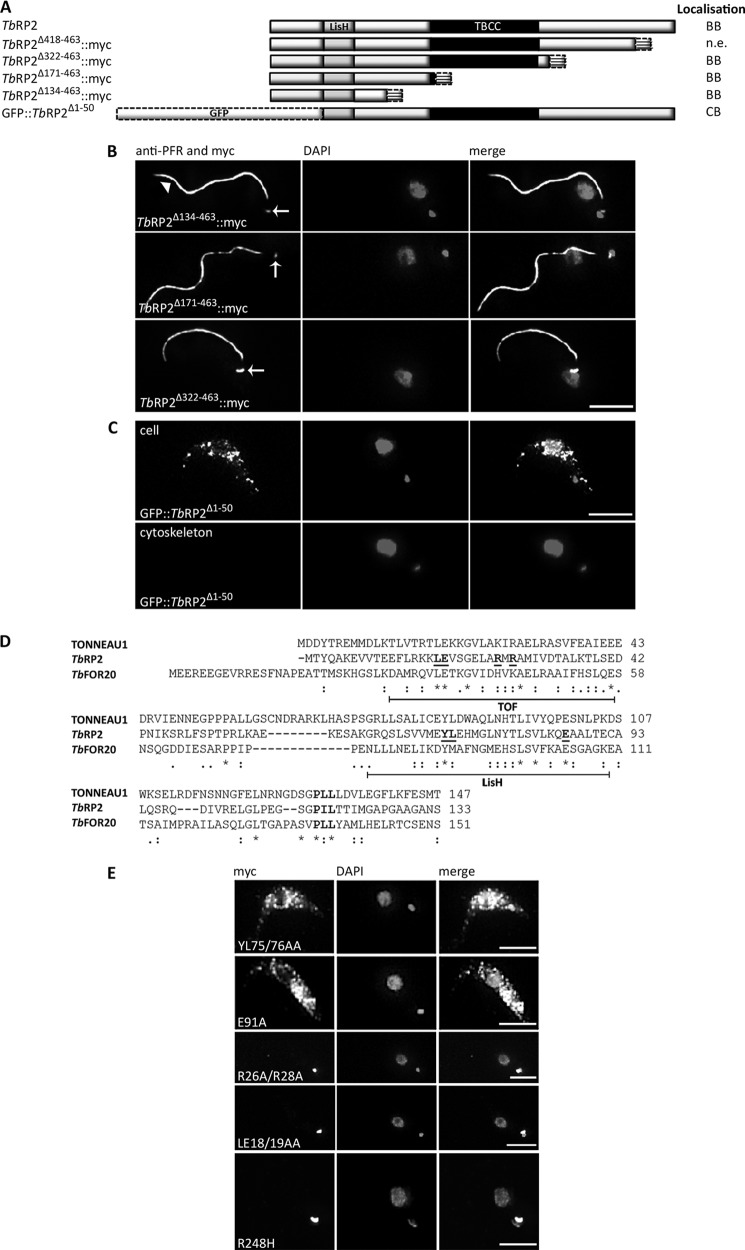
**Role of TOF/LisH motifs in targeting and tethering of *Tb*RP2.** *A*, schematic architectures and localization of C- and N-terminally deleted proteins generated in this study. *BB*, basal body; *CB*, cell body; *n.e.*, no expression. *B*, immunofluorescent localization of C-terminally deleted *Tb*RP2 mutant proteins in detergent-extracted *T. brucei* cells. myc-tagged *Tb*RP2 proteins were detected using anti-myc antibody (the *arrows* indicate localization of myc-tagged proteins at the basal body). Cytoskeletons were colabeled with another IgG monoclonal antibody, L8C4, a well characterized monoclonal antibody specific for the PFR (an extra-axonemal structure within the *T. brucei* flagellum). Both anti-myc and L8C4 were detected by the same fluorophore-conjugated secondary antibody, but as the PFR only associates with the axoneme after the flagellum exits the cell body, the indirect L8C4 immunofluorescence signal was well separated from the basal body signals for myc-tagged *Tb*RP2 variants. The kinetoplast and nucleus were labeled by DAPI. *C*, cell body accumulation of detergent-soluble GFP::*Tb*RP2^Δ1–50^. *D,* sequence alignment of the N-terminal amino acid sequences of TONNEAU1 (34), *Tb*RP2, and the entire *Tb*FOR20 open reading frame. Amino acid identity (*asterisk*) and similarity (:) between all three sequences are indicated. Gaps (-) were inserted to maximize alignment. TOF and LisH motifs are delineated, and key amino acids subjected to site-directed mutagenesis are shown in *boldface* and *underlined. E*, localization of *Tb*RP2 variant proteins specifically altered by site-directed mutagenesis within the LisH (YL75/76AA and E91A) and TOF (R26A/R28A and LE18/19AA) motifs or the TBCC domain (R248H). *Scale bars* = 5 μm.

Because neither N- nor C-terminal epitope tagging affected TbRP2 localization or function (see subsequent “Results” section), we made a series of deletions to delineate the *Tb*RP2 amino acid sequences necessary for basal body targeting and tethering (Fig. 2*A*). Mutant proteins deleted from the C terminus were expressed with C-terminal myc tags (*Tb*RP2^Δ418–463^::myc, *Tb*RP2^Δ322–463^::myc, *Tb*RP2^Δ171–463^::myc, and *Tb*RP2^Δ134–463^::myc). Following a series of independent stable transformations, we never saw expression of the mutant *Tb*RP2^Δ418–463^::myc protein, presumably because of problems with protein stability. This truncation deleted a C-terminal region which in all trypanosomatids, and their free-living ancestor *Bodo saltans,* is rich in acidic amino acids. In contrast, deletion up to the centrally located TBCC domain (*Tb*RP2^Δ322–463^::myc), up to and including the TBCC domain (*Tb*RP2^Δ171–463^::myc), and even deletion up to a predicted N-terminal LisH motif (*Tb*RP2^Δ134–463^::myc) resulted in protein expression and basal body localization, which was retained in detergent-extracted cytoskeletons (Fig. 2*B*). N-terminal deletion of the first 50 amino acids, however, abrogated basal body localization and resulted in the accumulation of soluble tagged protein (GFP::*Tb*RP2^Δ1–50^) within the cell body (Fig. 2*C*). Preparation of cytoskeletons confirmed the absence of basal body localization for GFP::*Tb*RP2^Δ1–50^.

Inspection of the N-terminal sequence of *Tb*RP2 revealed that upstream of the predicted LisH motif lies a sequence related to the TOF motif defined recently from the comparison of TONNEAU1 homologs from plants (which function in dynamic cortical cytoskeleton organization) with the centrosomal proteins OFD1 (oral-facial-digital syndrome 1), FOP (FGR1 oncogene partner), and FOR20 (FOP-related protein of 20 kDa) from animals ((36, 37, Fig. 2*D*). Focusing on residues conserved in different LisH motifs or between the predicted TOF motifs in *Tb*RP2 and Tonneau1 homologs, we performed four site-directed mutagenesis reactions (YL75/76AA, E91A, LE18/19AA, and R26A/R28A). Both mutations within the LisH motif abolished basal body targeting and resulted in accumulation of protein in the cell body. In contrast, our mutations within the TOF motif affected neither protein targeting nor basal body tethering (Fig. 2*E*). However, the results from the short N-terminal deletion (Fig. 2*C*) nonetheless indicate a requirement for the TOF motif *per se* for *Tb*RP2 localization. Collectively, these results suggest that information necessary to both target and tether *Tb*RP2 at the transitional fibers of mature flagellar basal bodies is contained within the N-terminal 133 amino acids.

##### Mutation of Arg-248 Abolishes TbRP2 Function

**FIGURE 3.**
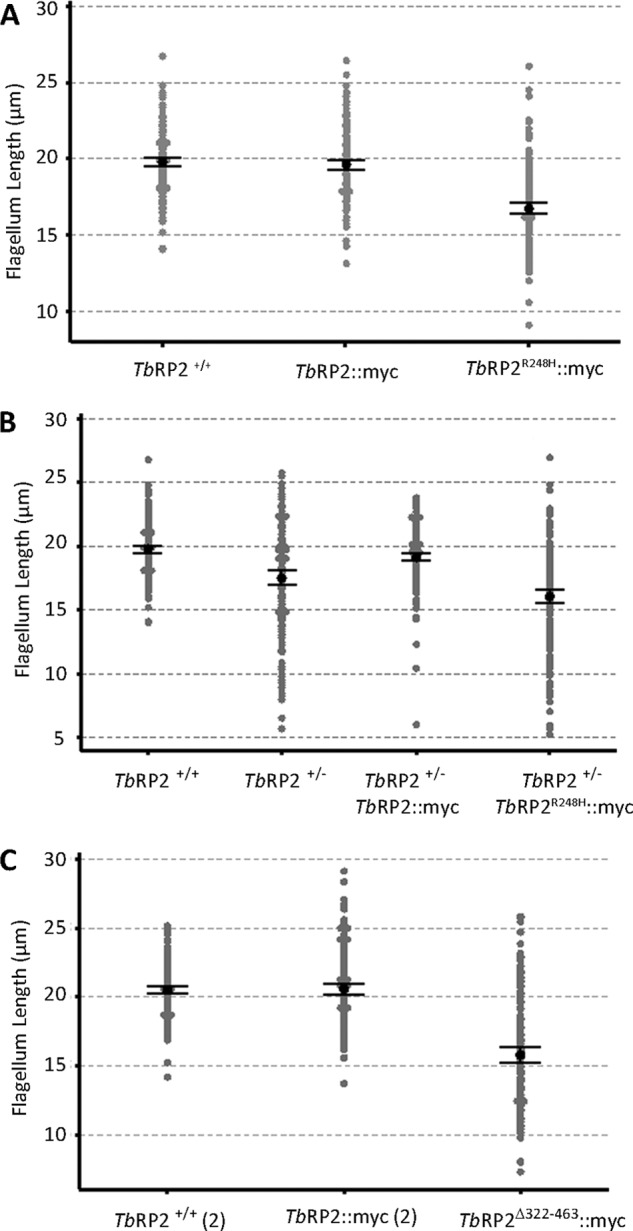
**Effect of Tb*RP2* mutation on flagellum length.** Flagellum length was measured in either wild-type parental Tb*RP2*^+/+^ or heterozygous Tb*RP2*^+/−^ cells expressing recombinant myc-tagged *Tb*RP2 variants (*n* = 200 for each analysis). The genetic background for myc-tagged protein expression was Tb*RP2*^+/+^ unless indicated otherwise. Individual data points are shown as *gray circles*, and *black circles* indicate the mean with associated 95% confidence intervals. Comparisons of flagellum length between populations were statistically evaluated using the non-parametric Mann-Whitney *U* test. Non-significant *p* values of 0.351 and 0.405 were returned when comparing *Tb*RP2^+/+^ with *Tb*RP2::myc (*A*) and *Tb*RP2^+/+^ with *Tb*RP2::myc (2) (*C*), respectively. All other comparisons were deemed significantly different (*p* = < 0.05) with *p* values of *p* = 0.000 returned in all cases, except when comparing *Tb*RP2^+/+^ with *Tb*RP2^+/−^ *Tb*RP2::myc (*B*) (*p* = 0.013).

We stably transformed diploid *T. brucei* with constructs yielding constitutive ectopic expression of *Tb*RP2::myc or a mutant protein (*Tb*RP2^R248H^::myc) in which mutation of the analogous arginine in canonical TBCC and XRP2 abolishes physiological function in tubulin processing because of the loss of GAP activity (16). Expression of *Tb*RP2::myc had little effect on flagellum length, whereas expression of *Tb*RP2^R248H^::myc gave a dominant negative phenotype of significantly reduced average flagellum length (Fig. 3*A*). We then took advantage of our observation that deletion of a single *TbRP2* allele in diploid *T. brucei* resulted in a haploid insufficiency phenotype, characterized by significant heterogeneity in flagellum length (Fig. 3*B*). In *T. brucei*, flagellum length is also a critical determinant of cell body length (38), and so TbRP2^+/−^ cells also exhibit morphological heterogeneity. However, deletion of an endogenous *TbRP2* allele on the background of ectopic *Tb*RP2::myc overexpression did not phenocopy the haploid insufficiency phenotype, indicating functionality of myc-tagged *Tb*RP2. Failure to observe haploid insufficiency-like phenotypes in cells expressing either GFP::*Tb*RP2 or *Tb*RP2::GFP indicates that these mutant proteins were also functional. In contrast, *TbRP2* allele deletion on a background of *Tb*RP2^R248H^::myc expression resulted in cell populations exhibiting even more marked reductions in flagellum length than seen in Tb*RP2*^+/−^ populations (Fig. 3*B*), indicating an essentiality of a predicted active-site arginine within the TBCC domain of *Tb*RP2 and that *Tb*RP2 exerts its biochemical function as a basal body-localized GAP. The R248H mutation did not influence protein localization (Fig. 2*E*). The phenotypes resulting from expression of *Tb*RP2^R248H^::myc point toward the probability of dominant negative effects on flagellum formation. A similar dominant negative phenotype was seen following expression of the *Tb*RP2^Δ322–463^::myc mutant on a wild-type diploid background (Fig. 3*C*), but neither *Tb*RP2^Δ134–463^::myc nor *Tb*RP2^Δ171–463^::myc expression resulted in a significant change in cell morphology or flagellum length (data not shown).

##### Failure to Recruit Ciliary Gate Components in TbRP2 RNAi Mutants

**FIGURE 4.**
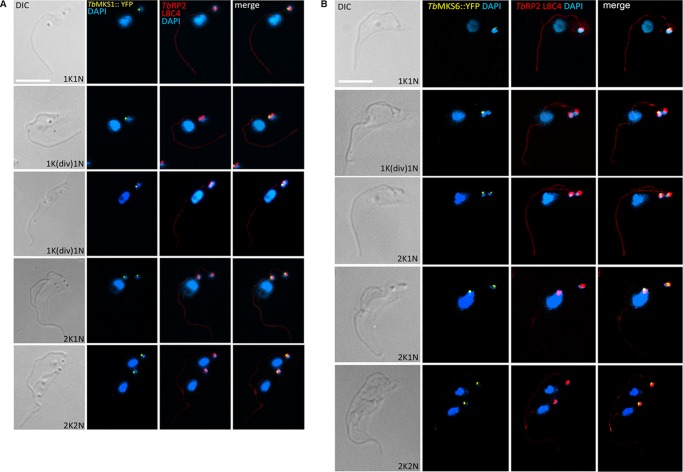
**Recruitment of candidate transition zone orthologs to the trypanosome flagellum.** *A*, cell cycle-dependent accumulation of *Tb*MKS1::YFP to a focus distal to the transitional fibers of the mature basal body. Detergent-extracted cells were stained for cofluorescence with monoclonal antibody L8C4 (to detect the PFR) and anti-*Tb*RP2 antibodies. *Tb*MKS1::YFP incorporation into the new flagellum occurs following basal body duplication (*1K(div)1N*). *DIC,* differential interference contrast. *B*, cell cycle-dependent accumulation of *Tb*MKS6::YFP to a focus distal to the transitional fibers of the mature basal body. Detergent-extracted cells were stained for cofluorescence with monoclonal antibody L8C4 (to detect the PFR) and anti-*Tb*RP2 antibodies. *Tb*MKS6::YFP incorporation into the new flagellum occurs following basal body duplication (*1K(div)1N*) and initiation of IFT-dependent flagellum elongation (as detected by L8C4 labeling of the new flagellum in the *second panel* (*1K(div)1N*)). *Scale bars* = 5 μm.

Localization of RP2 proteins to transitional fibers at the mature basal body means that RP2 is a component of the ciliary gate that, together with the TZ, regulates flagellum protein content (11). Protein complexes within the TZ contributing to this gating function have recently begun to be described in molecular terms and include widely conserved gene products that, when defective in human cells, give rise to inherited ciliopathies (8). We recently characterized candidate *T. brucei* TZ components, including trypanosome orthologs of MKS1 and MKS6,^4^ which are subunits of a large (TZ-located) MKS complex (MKS (MIM249000), also known as Meckel-Gruber syndrome, is a lethal autosomal recessive ciliopathy) (39, 40). YFP-tagged *Tb*MKS1 (*Tb*MKS1::YFP^Ty^) and *Tb*MKS6 (*Tb*MKS6::YFP^Ty^) localize distal to *Tb*RP2, consistent with both *Tb*MKS1 and *Tb*MKS6 localizing to the TZ in the trypanosome flagellum (Fig. 4, *A* and *B***)**. At the beginning of the cell division cycle, the trypanosome cell possesses a single basal body (nucleating a single flagellum) with an immature (probasal) body lying immediately alongside. Such cells display a single focus of *Tb*RP2, *Tb*MKS1, and *Tb*MKS6. As the trypanosome enters the cell cycle, however, the probasal body matures into a basal body that nucleates a second flagellum. This basal body maturation event is marked by recruitment of *Tb*RP2 to the newly matured basal body. Thus, these cells show two foci of *Tb*RP2. Interestingly, careful examination of early basal body maturation events reveals that, in *T. brucei*, a newly matured basal body can be *Tb*RP2-positive (and also have a nucleated a short new flagellum) prior to acquisition of *Tb*MKS1 and *Tb*MKS6 (Fig. 4, *A* and *B*), at least as revealed by the sensitivity of YFP fluorescence detection. As the new flagellum elongates, *Tb*MKS1 and *Tb*MKS6 proteins are subsequently recruited to the proximal region of the new flagellum.

**FIGURE 5.**
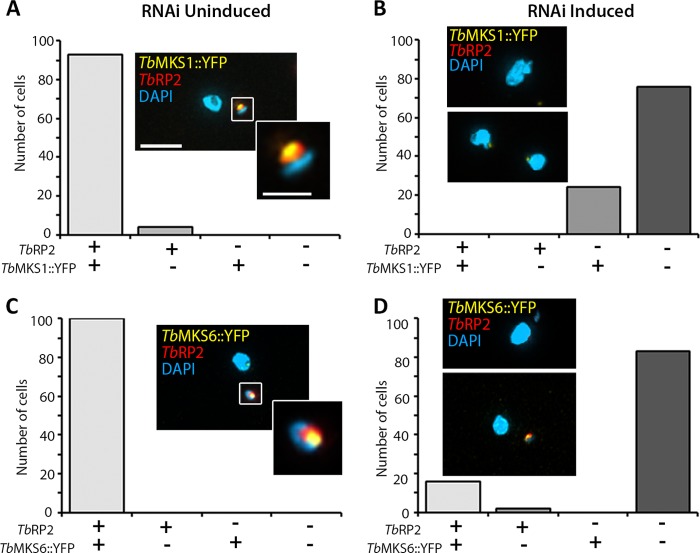
**Effect of Tb*RP2* RNAi on the recruitment of transition zone proteins.** Immunofluorescence images of detergent-extracted cells showing that *Tb*MKS1::YFP^Ty^ and *Tb*MKS6::YFP^Ty^ proteins (which normally localize to the proximal region of the axoneme distal to *Tb*RP2 (see Fig. 4, *A* and *B*)) fail to recruit to this region following induction of TbRP2 RNAi. The *boxed areas* in the *merged panels* are enlarged in the *insets. Main panel scale bar* = 5 μm; *inset scale bar* = 2 μm. Graphs indicate the percentage of cells positive for *Tb*RP2 and either *Tb*MKS1::YFP^Ty^ or *Tb*MKS6::YFP^Ty^ expression following TbRP2 RNAi induction. Approximately 20% of cells remained weakly positive for *Tb*MKS1::YFP^Ty^ expression in the absence of *Tb*RP2, whereas ∼15% of cells remained both *Tb*MKS6::YFP^Ty^- and *Tb*RP2-positive. All images were collected using identical acquisition parameters.

Following RNAi-mediated ablation of *Tb*RP2, the probasal body matures to nucleate a new flagellum (albeit one that is ultimately short and structurally defective) but is devoid of *Tb*RP2 protein. Interestingly, we observed that both *Tb*MKS1::YFP and *Tb*MKS6::YFP also failed to localize to the TZ in Tb*RP2* RNAi-induced cells (Fig. 5, *A* and *B*). In immunoblot analysis of whole cell extracts, *Tb*MKS1::YFP^Ty^ could not be detected following induction of Tb*RP2* RNAi (data not shown), suggesting that, if *Tb*MKS1 fails to recruit to the TZ, it is targeted for degradation. RP2-dependent localization of MKS1 or MKS6 homologs was unexpected and speaks directly to the debate regarding the role of RP2 in cilium formation/function.

##### TbRP2 Contains the YL1/2 Epitope

**FIGURE 6.**
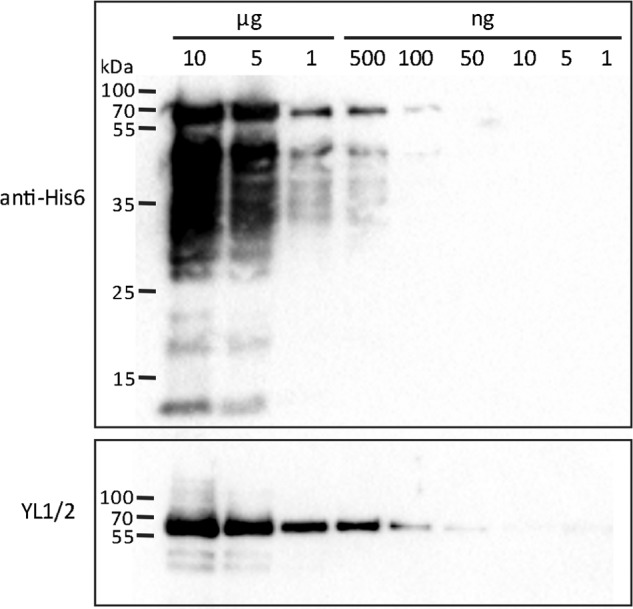
***Tb*RP2 is recognized by the monoclonal antibody YL1/2.** Immunoblot analyses showing detection of decreasing amounts of recombinant *Tb*RP2 protein containing an N-terminal hexahistidine tag detected with an anti-His_6_ monoclonal antibody and the monoclonal antibody YL1/2. Differences in the immunoblot signals reflect the specificity of YL1/2 for a C-terminal DDY epitope *versus* detection of full-length and truncated recombinant proteins (either as a consequence of premature translation termination or proteolytic degradation) with the anti-His_6_ antibody.

Failure to recruit *Tb*MKS1 and *Tb*MKS6 to the TZ following TbRP2 RNAi induction led us to re-evaluate how loss of *Tb*RP2 gives rise to defects in flagellum assembly. We reported previously (and again confirm in this study) that TbRP2 RNAi induction results in specific loss of YL1/2 labeling at the basal body. Loss of YL1/2 reactivity is rapid and equally affects both the new and old basal body in biflagellate cells (17). This was interpreted as evidence that loss of *Tb*RP2 from the trypanosome basal body affects recruitment/processing of tubulin destined for axonemal incorporation. However, detection of a pool of α-tubulin at the transitional fibers of the trypanosome basal body by YL1/2 is puzzling, not least because other anti-tubulin antibodies (*e.g.* the anti α-tubulin monoclonal antibody TAT1 (41)) label only the core microtubule structure of the trypanosome basal body. However, the YL1/2 antibody recognizes a linear epitope defined as a carboxy-terminal aromatic residue preceded by two negatively charged amino acids (42), and, in revisiting RP2 function, we noted that *Tb*RP2 ends with a classic YL1/2 recognition epitope (ending DDF). Moreover, in their study, Wehland *et al.* (42) showed that substitution of phenylalanine for tyrosine considerably enhanced YL1/2 antigenic reactivity, suggesting that *Tb*RP2 could provide a better antigen than trypanosome α-tubulin (ending EEY). Indeed, immunoblot analysis of recombinant *Tb*RP2 protein showed efficient recognition by YL1/2. Specificity of YL1/2 for full-length protein was confirmed by the detection of an identical immunoblot with anti-His_6_ monoclonal antibody, which detected the N-terminal His_6_-tag present on full-length protein and a variety of premature translation termination and/or C-terminally degraded proteolytic products (Fig. 6). Efficient recognition of *Tb*RP2 by YL1/2 must, therefore, strongly challenge our previous interpretation regarding TbRP2 RNAi-induced loss of YL1/2 labeling at the basal body and our understanding of *Tb*RP2 function in trypanosome flagellum biogenesis.

##### Human XRP2 Does Not Compensate for Loss of TbRP2 from the Trypanosome Basal Body

**FIGURE 7.**
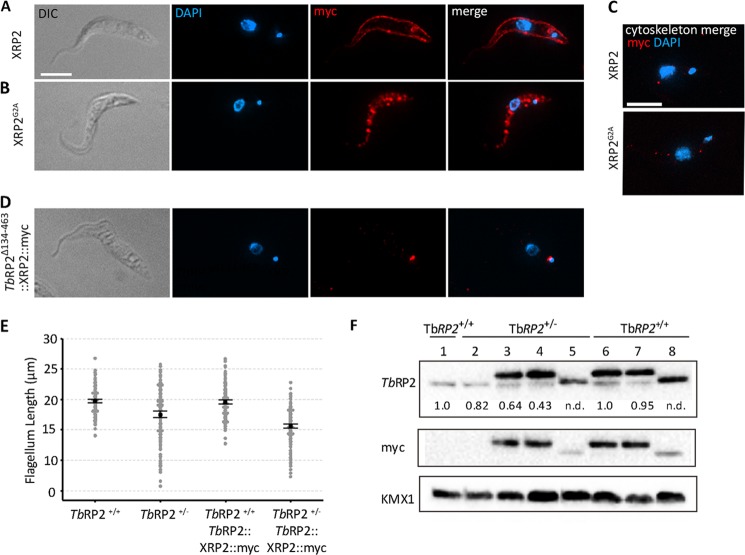
**Expression of human XRP2 in *T. brucei*.** *A*, indirect immunofluorescence analysis of trypanosome cells expressing XRP2::myc. *Scale bar* = 5 μm. *DIC,* differential interference contrast. *B*, indirect immunofluorescence analysis of trypanosome cells expressing XRP2^G2A^::myc. XRP2::myc localizes to the plasma membrane, whereas mutation of the glycine target for myristoylation results in cytoplasmic XRP2 accumulation. *C*, preparation of detergent-extracted cytoskeletons confirms the absence of basal body association for either protein. *Scale bar* = 5 μm. *D*, indirect immunofluorescence shows mature basal body localization of a chimeric *Tb*RP2^Δ134–463^::XRP2::myc protein. *E*, flagellum length measurements in either wild-type parental Tb*RP2*^+/+^ or heterozygous Tb*RP2*^+/−^ cells expressing *Tb*RP2^Δ134–463^::XRP2::myc (*n* = 200 for each analysis). Statistical analysis was as described in Fig. 3. Significant *p* values of 0.000 were returned for all comparisons, except when comparing *Tb*RP2^+/−^ with *Tb*RP2::XRP2::myc (*p* = 0.360). *F*, immunoblot analysis of myc-tagged *Tb*RP2- and *Tb*RP2^Δ134–463^::XRP2::myc-expressing cell lines used for flagellum length measurements shown in Figs. 3 and 7*E. Lanes 1* and *2*, cells not transfected with constructs for expression of myc-tagged protein; *lane 3*, *Tb*RP2::myc; *lane 4*, *Tb*RP2^R248H^::myc; *lane 5*, *Tb*RP2^Δ134–463^::XRP2::myc; *lanes 6* and *7*, *Tb*RP2::myc; and *lane 8*, *Tb*RP2^Δ134–463^::XRP2::myc. 5 × 10^6^ cell equivalents were loaded per lane. *Tb*RP2^Δ134–463^::XRP2::myc migrates with the same apparent molecular weight as native *Tb*RP2. In the blot probed with anti-*Tb*RP2 antibodies, the *lower band* detects native *Tb*RP2. The intensity of *Tb*RP2 detected with anti-*Tb*RP2 antibodies relative to the intensity observed in the wild-type background (*lane 1*) is shown, except in cell lines expressing *Tb*RP2^Δ134–463^::XRP2::myc, where comigration with native *Tb*RP2 precludes this analysis. Normalization was made using KMX1 loading control and the ChemiDoc-associated software Image Lab 4.0 (Bio-Rad).

Human XRP2 and TBCC have overlapping biochemical functions and partially complement the microtubule phenotype resulting from deletion of the TBCC homolog in yeast (16). We asked whether heterologous expression of human XRP2 in *T. brucei* could complement the loss of *Tb*RP2 expression. In human cells, XRP2 is a myristoylated protein found at multiple locations, including the basal body, a localization dependent upon myristoylation (23). Heterologous expression in *T. brucei* of XRP2 with a C-terminal myc tag resulted in XRP2::myc accumulation only at the plasma membrane (Fig. 7*A*). Site-directed mutagenesis of the glycine target for myristoylation, followed by expression of the resulting XRP2^G2A^::myc protein, resulted in accumulation of soluble protein in the trypanosome cytosol, but again, protein was not located at the basal body (Fig. 7, *B* and *C*). We then asked whether XRP2 could be artificially targeted to the mature basal body in trypanosomes if it was fused to the N-terminal 133 amino acids of *Tb*RP2, which, as we had shown, were necessary and sufficient for basal body recruitment of *Tb*RP2. Constitutive expression of this chimeric protein (*Tb*RP2^Δ134–463^::XRP2::myc) resulted in faithful localization to mature basal bodies. That is, protein did not accumulate in the cytosol or at other sites within the cell body (Fig. 7*D*). Moreover, *Tb*RP2^Δ134–463^::XRP2::myc was also retained at basal bodies in cytoskeletons, confirming a stringent association with the mature basal body. Curiously, although *Tb*RP2^Δ134–463^::XRP2::myc expression had little effect on flagellum length in a wild-type Tb*RP2*^+/+^ background, in a Tb*RP2*^+/−^ background, the average flagellum length was reduced in much the same way that expression of *Tb*RP2^R248H^::myc exacerbated flagellum length reduction of a haploid insufficiency mutant (Fig. 7*E*). Immunoblot analysis indicated that the dominant negative phenotype arising from *Tb*RP2^Δ134–463^::XRP2::myc expression arose despite a steady-state accumulation of less myc-tagged protein than was observed for experiments in which either *Tb*RP2::myc or *Tb*RP2^R248H^::myc were expressed (Fig. 7*F*).

## DISCUSSION

Given the data available in the literature, our interests were to resolve how *Tb*RP2 is targeted and tethered to mature basal bodies in *T. brucei* and to reconcile alternative hypotheses with regard to RP2 function in ciliogenesis. Insight gleaned from our studies is discussed below.

Deletion from N and C termini indicated that N-terminal TOF and LisH motifs were sufficient for basal body targeting of *Tb*RP2. Curiously, the co-option of TOF and LisH motifs for targeting an RP2 protein appears to be unique to trypanosomatids and their free-living protist ancestor *B. saltans* (trypanosomatids and *B. saltans* are both part of the Kinetoplastid order), as to date we have not found TOF-LisH motifs in conjunction with a TBCC domain in any other eukaryote, including *Chlamydomonas* and ciliates that represent other genetically tractable model flagellates. Kinetoplastid RP2 proteins, therefore, increase the number of microtubule organizing center (MTOC)-associated proteins that possess N-terminally located TOF and LisH motifs. Of these other proteins, FOP, OFD1 and FOR20 are widely conserved in flagellate eukaryotes, and Tonneau 1 is conserved in acentriolar land plants, where it is required for microtubule-based cytoskeletal organization. Why co-option of TOF-LisH motifs to a TBCC domain occurred during trypanosome evolution is not obvious, but regarding the role of TOF-LisH motifs in conferring basal body localization, the motifs from *Tb*RP2, mammalian FOP (43), and FOR20 (37, 44) provide a single targeting determinant. Yet, in the case of OFD1, site-directed mutation of the LisH motif has yet to establish the importance of the motif in conferring localization. Indeed, if the OFD1 LisH motif does function in localization, it must do so in conjunction with downstream coiled coil regions that are required for centrosomal localization of human OFD1 (45, 46).

Site-directed mutagenesis within the LisH motif identified residues conserved widely between LisH motifs that were necessary for *Tb*RP2 targeting. Classically, the LisH motif is thought of as a motif for dimerization of diverse proteins found at many sites inside cells (47). Site directed mutations within the *Tb*RP2 LisH motif which affect basal body targeting may therefore be abrogating vital protein-protein interactions between *Tb*RP2 and other proteins required for basal body localization. The identity of these partner proteins remains cryptic at present, but we have characterized *T. brucei* orthologs of FOP, OFD1 and FOR20 and find no evidence that *Tb*RP2 localization is compromised and there is no phenotype convergence when each trypanosome TOF-LisH motif-containing protein is targeted by gene-specific RNAi.^5^ The available evidence, thus, suggests that the small cohort of TOF-LisH motif-containing proteins conserved in flagellate eukaryotes do not come together to form a functional complex. Although it is interesting to question why TOF-LisH motifs are so sparingly used to facilitate targeting of only a handful of proteins required for the assembly of mature basal bodies and their associated appendages (37, 43, 44, 46), this is beyond the scope of this study.

How does *Tb*RP2 function in flagellum assembly? The effect of the R248H mutation on flagellum length indicates that *Tb*RP2 is a *bona fide* GAP, but the question to resolve is whether it is a GAP for tubulin (11, 17) or for another protein that functions more generally in protein trafficking and flagellum assembly/function, as postulated for XRP2 in animal cells (20). The initial characterization of *Tb*RP2 function predated the discovery of a modular MKS complex in mammalian cilia and the inference of a similar complex in *Chlamydomonas* regulating the accumulation of both membrane-associated and soluble flagellar proteins (40, 48). Here, we report that, following TbRP2 RNAi induction, orthologs of MKS6 and MKS1 fail to be recruited to the proximal region of the trypanosome flagellum at a site congruent with TZ localization. Although failure to recruit or fully assemble the modular MKS complex could be explained as a consequence of microtubule-based defects (CEP162 is the conserved protein identified as mediating MKS complex association with axonemal microtubules (49)), our new data must lead to the consideration that both axonemal defects and the failure of *Tb*MKS6 and *Tb*MKS1 recruitment are consequences of broader defects in GAP-dependent protein processing, *i.e.* akin to the proposed role of XRP2 in ciliogenesis. However, the recent report that yeast TBCC has dual functionality, acting as a GAP for both tubulin and Alp41 (ortholog of human Arl2) (50), suggests that it is nonetheless prudent to consider whether RP2 may also have multiple roles in flagellum formation. In that regard, in the initial characterization of the TbRP2 RNAi-induced phenotype, great significance was attached to the specific loss of YL1/2 labeling at the basal body. This was taken as direct evidence that *Tb*RP2 functions in the processing/recruitment of tubulin destined for flagellum incorporation (17). Although YL1/2 is extensively used as a marker for tyrosinated α-tubulin, the epitope recognized by this monoclonal antibody is also encoded by other proteins (42, 51), including, as we reveal in this study, *Tb*RP2. Thus, as *Tb*RP2 is also a target for YL1/2 detection, critical experimental evidence used to support the claim for *Tb*RP2 acting in a tubulin processing capacity is now open to question. It is possible (indeed likely) that loss of basal body YL1/2 labeling in TbRP2 RNAi-induced cells reflects depletion of *Tb*RP2 rather than consequent loss of α-tubulin. As *Tb*RP2 and α-tubulin are of near identical molecular mass and isoelectric point (α-tubulin, 50.6 kDa; pI, 4.6 and *Tb*RP2, 49.8 kDa; pI 4.7), we cannot differentiate between these two proteins in immunoblotting experiments and, thus, are unable to confirm loss of YL1/2-reactive *Tb*RP2 following the induction of TbRP2 RNAi. Nevertheless, the re-evaluation of the possible target of YL1/2 at the basal body, together with the demonstration that TbRP2 RNAi induction perturbs the recruitment of TZ proteins, suggests that *Tb*RP2 may function in the regulation of protein trafficking to the trypanosome basal body (*i.e.* akin to the role proposed for XRP2) rather than in a dedicated tubulin processing/quality control capacity, as suggested previously.

In accommodating both possible models for *Tb*RP2 function (*i.e.* in general protein trafficking *versus* general protein trafficking and tubulin folding), the studies on mammalian cells have shown that siRNA-mediated knockdown of proteins forming the B9 complex (which is located at the TZ and contains both MKS1 and MKS6) affects ciliogenesis in certain cell types (*e.g.* inner medullary collecting duct cells and IMCD3 cells) (39). However, as siRNA ablation of B9 complex proteins does not affect ciliogenesis in hippocampal neurons, the effect of TZ disruption appears to be tissue/cell-specific. It is suggested that ciliary defects may be more severe in rapidly growing tissues/cells (*e.g.* IMCD3 cells) than in differentiated, non-proliferative cells such as neurons (39). In IMCD3 cells, siRNA-mediated knockdown of TZ proteins reduces both the number and length of cilia, but as IFT particles are readily detected in shortened cilia, the defect in ciliogenesis is not due to a failure in IFT. Similarly, the TbRP2 RNAi phenotype is distinctive from the RNAi phenotypes arising from loss of either anterograde or retrograde IFT components (52, 53). Thus, the short flagellum phenotype of TbRP2 RNAi cannot simply be explained by a failure in IFT.

Finally, work in animal systems points to IFT-independent assembly of the TZ prior to axoneme elongation (11, 39). In contrast, our work indicates that MKS1 and MKS6 recruitment, and, by inference, TZ maturation, occurs after flagellum elongation has begun. Indeed, for at least *Tb*MKS6, recruitment is detectable only after PFR assembly has commenced. This occurs after the flagellum has extended ∼2 μm and exited from the cell via a flagellar pocket. The recent characterization of a trypanosomatid-specific protein, KHARON1 (in the closely related trypanosomatid parasite *Leishmania mexicana*) indicates that ciliary gate function in trypanosomes may involve both conserved and lineage-specific mechanisms (54). In a wider cellular context, perhaps one question is whether either trypanosomes or animals represent exceptions with regard to the timing of TZ assembly during ciliogenesis.

## Footnotes

4 X. Qi, J. Andre, M. L. Ginger, and P. G. McKean, unpublished observations.

5 J. Andre, M. L. Ginger, and P. G. McKean, unpublished observations.

## Footnotes

3 The abbreviations used are:

IFT: intraflagellar transport
TZ: transition zone
TBC: tubulin cofactor
TBCC: tubulin cofactor C
GAP: GTPase-activating protein
MKS: Meckel syndrome
PFR: paraflagellar rod.
